# Antibacterial Efficacy of Calcium Hydroxide and Chlorhexidine Mixture for Treatment of Teeth with Primary Endodontic Lesions: A Randomized Clinical Trial

**DOI:** 10.22037/iej.2016.1

**Published:** 2016

**Authors:** Zakiyeh Donyavi, Parastoo Ghahari, Mohammad Esmaeilzadeh, Mohammadjavad Kharazifard, Rasoul Yousefi-Mashouf

**Affiliations:** a *Department of Endodontics, Dental School, Hamadan University of Medical Sciences, Hamadan, Iran; *; b * Department of Pediatric Dentistry, Dental School, Hamadan University of Medical Sciences, Hamadan, Iran; *; c * Dental Research Center, Dentistry Research Institute, Tehran University of Medical Sciences, Tehran, Iran; *; d *Department of Medical Microbiology, Hamadan University of Medical Sciences, Hamadan, Iran*

**Keywords:** Calcium Hydroxide, Chlorhexidine, Endodontic Treatment, Periapical Abscess, Root Canal Therapy

## Abstract

**Introduction:**

This study compared the root canal microbial count of necrotic teeth after irrigation with 6% sodium hypochlorite (NaOCl) (single session treatment) and two-session root canal treatment with two-week application of calcium hydroxide (CH) mixed with 0.2% chlorhexidine (CHX) as intracanal medicament.

**Methods and Materials:**

In this randomized clinical trial, single-rooted necrotic teeth were divided into two groups. Root canal was irrigated with 2 mL of 6% NaOCl in one group, and a mixture of 0.2% CHX and CH powder as an intracanal medicament for two weeks, in the other group. Root canal samples were obtained before and after the intervention and number of colony forming units (CFUs) was counted in each phase.

**Results:**

The reduction of *Enterococcus faecalis* CFU was not significantly different between the two groups (*P*=0.233) but the CFU of aerobic and anaerobic bacteria was significantly lower in CH+CHX group (*P*<0.001).

**Conclusion:**

Two-week application of CH+CHX caused significant reduced the aerobic, anaerobic and *E. faecalis *colony counts. Thus, it may be beneficial to carry out primary root canal treatment of necrotic teeth with endodontic lesions in two sessions with intracanal medicaments to achieve predictable results.

## Introduction

Bacteria and their products are the main cause of pulpitis and periradicular lesions [1, 2]. The primary goal of endodontic therapy is to clean and disinfect the root canal system and provide seal against the invasion and reentry of bacteria. Thus it can be stated that success of endodontic treatment highly depends on the prevention of bacterial proliferation, creation of a seal and prevention of re-infection [3]. 

Of the different materials, sodium hypochlorite (NaOCl) is the most effective irrigating solution in terms of antimicrobial activity and dissolution of organic debris. In teeth with periapical lesions that were infected with *Enterococcus faecalis* (*E. faecalis*) and required re-treatment, two-week application of calcium hydroxide (CH) and chlorhexidine (CHX) as an intracanal medicament significantly decreased the count of this resistant microorganism [4]. A clinical trial by Vera *et al.* [5], compared the microbiota of root canals with primary apical periodontitis that had undergone one or two-session treatments. They demonstrated that none of the teeth in single-session treatment group were completely free from bacteria and microorganisms were found in the main canal path, isthmii, apical foramen and dentinal tubules. Of the teeth in the two-session treatment group, two were completely free from bacteria. Bacteria in the main canal path were seen in only two samples and the residual bacteria in the isthmii and apical foramen were much lower than those in the one-session group. 

Controversy still exists regarding the treatment of certain conditions in endodontics. Single-session root canal treatment of the teeth with periapical lesions is among the controversial topics. In such cases, single-session endodontic treatment would be ideal if the pulp is vital due to the low bacterial count in the canal. If performed aseptically, the treatment can be accomplished within a single session. However, there are concerns regarding single-session biomechanical preparation and immediate obturation of the root canals of teeth with necrotic pulps and radiographically confirmed periapical lesions, because the adequacy of cleaning the root canal system cannot be ensured. 

It appears that application of a mixture of CH and CHX as an intracanal medicament for two weeks increases the success rate of re-treatment in teeth with periapical lesions. Application of intracanal medicaments can significantly decrease the count of intracanal microorganisms. Thus, this strategy may be useful for primary treatment of teeth with periapical lesions, in order to decrease the risk of reinfection. Otherwise, by using a strong antimicrobial agent, the treatment can be accomplished within a single session. Patient’s preference for a single or two-session treatment must be considered as well; since most patients prefer two short sessions over one long session. This study aimed to compare the efficacy of single session treatment with the application of 6% NaOCl with two-session treatment using 6% NaOCl and two-week intracanal medication with CH and CHX for treatment of teeth with primary periapical lesions.

## Materials and Methods

After approval by the Ethics Committee of Hamadan University, School of Dentistry (Grant No.: P/16/35/9/5973), this randomized clinical trial was registered at www.irct.ir (identification No.: IRCT201502169014N55) and was performed according to the declaration of Helsinki. Patients presenting to Endodontic department of Hamadan University, were clinically examined by the researcher and those with a single-rooted, necrotic teeth with chronic apical periodontitis were selected for further evaluations. All phases of the study were conducted by a single operator for quality control purposes. A total of 30 single-rooted teeth with necrotic pulps were selected for this study (Figure 1). The teeth all responded negatively to pulp test and had a periapical lesion confirmed on periapical radiographies. Patients with a history of antibiotic therapy in the past three months, extensive caries, crown/root fracture, history of previous root canal therapy and deep pockets (>4 mm) were excluded. 

The possible complications and risks of treatment were thoroughly explained to patients and all patients signed written informed consent forms before the beginning of the treatment. 

After administration of local anesthesia using 2% lidocaine containing 1:80000 epinephrine (Darupakhsh, Tehran, Iran), supragingival scaling was performed. Prophylaxis was carried out with pumice paste and rubber dam isolation was done. Caries and coronal restorations were removed using sterile burs. The areas around teeth were disinfected with 30% hydrogen peroxide and 2.5% NaOCl. 

Following access cavity preparation, the area adjacent to the teeth was disinfected for the second time as described earlier. Next, 5% sodium thiosulfate was used to neutralize the effect of remaining NaOCl on tooth surfaces [6]. The specimens were randomly divided into two groups (*n*=15). The first sample (S1) was obtained from the root canal using a sterile #15 paper point. The height of paper point was 1 mm short of the radiographic apex in order to absorb the intracanal fluid [6]. Each paper point remained in the root canal for a minimum of 1 min. Paper points were then transferred into tubes containing 2 mL of thioglycolate broth for anaerobic bacteria and brain heart infusion (BHI, Merck, Darmstadt, Germany) broth for aerobic bacteria and *E. faecalis*. Working length was determined using an apex locator (Dentaport ZX, J. Morita Co., Kyoto, Japan) and later confirmed by periapical radiography. All canals were prepared with ProTaper rotary files (Dentsply Maillefer, Ballaigues, Switzerland) up to F3 (30/0.09) using the crown-down technique. Test group was selected randomly and the researcher and patients were blinded to the group allocation of samples. Specimens in the first group were coded with odd numbers while those in group two were coded with even numbers. 

In group 1, root canals of teeth were irrigated with 2 mL of 6% NaOCl using 5 mL syringes with 27-gauge needle between instruments (total irrigant volume=10 mL). Apical patency was maintained using #10 or 15 K-files (Dentsply Maillefer, Ballaigues, Switzerland). After completion of instrumentation, the root canals were irrigated with 2 mL of 5% sodium thiosulfate followed by 2 mL of saline solution. The second sample (S2) was obtained using #30 sterile paper points and transferred to the laboratory in the previously mentioned transfer mediums. Then root canals were filled with gutta-percha and AH-26 sealer (Dentsply, Tulsa Dental, Tulsa, OK, USA) using lateral compaction technique. The teeth were temporarily restored with Cavit (ESPE-Premier, Norristown, PA, USA) and the patient was referred for permanent tooth restoration. 

In group 2, all steps were done similar to group 1 and after irrigation with 6% NaOCl, a creamy mixture of 0.2% CHX (Shahrdaru, Tehran, Iran) and CH powder (Golchay, Tehran, Iran) was prepared and carried into the canal using a Lentulo spiral and packed into the root canal using a condenser [6, 7]. The crown was temporarily restored. The patients were recalled after 2 weeks and the second microbial sample (S2) was obtained using sterile #30 paper points after canal reopening and removing the intracanal medicament with saline irrigation [6]. The canals were then filled with gutta-percha and AH-26 sealer using cold lateral compaction technique. The teeth were temporarily restored and the permanent restoration was scheduled in the next session. Each paper point was immersed in 1 mL of thioglycolate (for anaerobes) and 1 mL of BHI broth (for aerobic bacteria and *E. faecalis*) transfer mediums and transferred to the lab within 30 min. 

In the lab, 1, 2, 5 and 10 μL of each medium (thioglycolate and BHI) were collected by a sampler and immersed in 1 mL of their respective medium type. Next, 10 μL was collected from each tube and transferred to a plate containing the respective medium. The collected sample from the thioglycolate medium was cultured on the Brucella agar enriched with defibrinated sheep blood and the sample collected from the BHI broth was cultured on kanamycin-esculin-azide agar and blood agar. Brucella agar plates were placed in an anaerobic jar. Using Gas-pak, an anaerobic environment was produced and the jar was incubated at 37^°^C for 72 h. Kanamycin plates were also incubated at 37^°^C for 48 h in the candle jar to allow proliferation of *E. faecalis*. Blood agar plates were also incubated at 37^°^C for 24 h to allow proliferation of aerobic bacteria. After completion of the respective time periods, plates were removed from the incubator and formed colonies were counted using a colony counter. The mean number of colony forming units (CFU) was reported based on the concentration of the primary dilute. Also, the magnitude of reduction in number of aerobic, anaerobic and *E. faecalis* colonies was calculated in each group and reported. 

The number of CFUs before and after treatment was described. The percentage of reduction in number of CFUs before and after the treatment in the two groups (irrigation with 6% NaOCl and irrigation plus application of a mixture of 0.2% CHX and CH) was analyzed and compared using the Mann Whitney U test because the distribution of the data was not normal (according to one-sample Kolmogorov-Smirnov test, *P*<0.05). The level of statistical significance was set at 0.05. All statistical analyses were performed using SPSS software (SPSS version 22.0, SPSS, Chicago, IL, USA).

## Results

The number and percentage of reduction of CFUs before and after the interventions are presented in Table 1. The percentage of reduction in *E. faecalis* count after the interventions was not significantly different between the two groups (*P*=0.233) but the percentage of reduction in aerobes and anaerobes was significantly higher in the second group (CHX+CH) compared to the first group (*P*=0.001). The mean reduction in aerobic, anaerobic and *E. faecalis* CFUs in group one (irrigation with 6% NaOCl alone), was 0.49±0.3%, 0.41±0.22% and 0.54±0.32%, respectively. In CH+CHX group, the mean reduction of CFU in aerobic, anaerobic and *E. faecalis* samples was 0.94±0.09%, 0.88±0.24% and 0.89±0.19%, respectively. 

## Discussion

This clinical trial compared the CFU of aerobic and anaerobic bacteria before and after single-visit (irrigation with 6% NaOCl) or double-visit (irrigation with 6% NaOCl and inter-appointment medication with CH+CHX) treatment of necrotic teeth with apical periodontitis. Based on the results of the current study, root canal preparation and irrigation in both groups caused significant reductions in aerobic, anaerobic and *E. faecalis* colony counts. 

Success or failure of endodontic treatment is determined *via* the conduction of clinical and radiographic examinations. However, for periapical lesions, due to the slow trend of healing, the treatment results may not be accurately assessed until five to 10 years after treatment [1]. Moreover, definition and calculation of indices related to radiographic examinations in periapical tissues are somehow problematic [8]. 

One immediate parameter for assessment of the success of endodontic treatment is assessment of the magnitude of reduction or elimination of microorganisms from the root canal system. Some studies have demonstrated that the treatment results of teeth with periapical lesions with negative culture at the time of root canal filling are more predictable [3, 9]. In general, elimination of microorganisms from the root canal is the main goal of endodontic treatment and may be considered as the ultimate goal in clinical trials [10].

Based on the results of the present study and CFU values, the magnitude of reductions was significantly greater in samples that were subjected to two-week application of CH+CHX mixture. Such superior antibacterial efficacy in this group is probably attributed to the application of intracanal medicament following irrigation with NaOCl and indicates the probable synergistic effect of CH and CHX. 

**Table 1.** Descriptive statistics [mean (SD)] of the number of CFUs in two groups

	**CFU before**		**CFU After**		**Percentage of reduction**	
	**Control**	**CHX**	**Control**	**CHX**	**Control**	**CHX**
**Aerobes**	17240 (22612)	29500 (26686)	7727 (6506)	888 (1444)	48.61 (30.41)	93.98 (8.83)
**Anaerobes**	24393 (22528)	40653 (28721)	15967 (17396)	1306 (6306)	40.05 (21.53)	87.71 (23.71)
***Enterococcus faecalis***	5400 (9700)	7846 (10154)	2457 (4525)	280 (1400)	63.19 (34.01)	88.99 (18.52)

On the other hand, previous studies have demonstrated that root canal preparation and irrigation with different solutions can significantly decrease the root canal microflora [11-13]. Similar to our findings, Lana *et al.* [14], reported that CH paste had a considerable effect on elimination of *E. faecalis* during mechanical and chemical preparation of the root canal system. Valera *et al.* [15], stated that although 2% CHX gel significantly decreased the microbial count, intracanal medicaments including CH and CH+CHX completely eliminated the microorganisms from the canals. Their findings confirmed the synergistic effects of CH and CHX. However, Delgado *et al.* [4], found no significant difference in terms of antibacterial properties of CHX gel with and without CH. Such controversy in the results may be attributed to the different culture techniques, root canal irrigating solutions, type of microorganisms and root canal anatomy. 

Based on the results of the current study, the frequency of anaerobic bacteria was significantly higher than aerobic bacteria and *E. faecalis* in both S1 (before treatment) and S2 (after treatment) samples; this finding has also been reported in some previous studies [8, 16, 17]. In the current study, all root canals before treatment contained bacteria; which indicates the strong correlation of presence of bacteria and periapical lesion in necrotic teeth. Figdor and Sundqvist [18] demonstrated that infected root canal system may contain up to 10^2^ to 10^8^ bacteria/mL; the values obtained in the current study are within this range as well. In a study by Kvist *et al.* [19], primary samples obtained from the specimens indicated the presence of microorganisms in 98% of the teeth [19]. 

NaOCl is a commonly used root canal irrigant. It has the highest efficacy as a root canal irrigating solution. Its clinical effects are attributed to its ability in dissolving the necrotic and organic tissues as well as its antibacterial properties [20]. On the other hand, recent clinical trials have indicated that 1.3% NaOCl is capable of eliminating the bacterial load by 95%; however, 30-40% of root canals showed signs of residual bacteria in the root canal system [21]. Mohammadi and Asgary showed that 6% NaOCl was effective against *Candida albicans* as well [22]. 

Abbaszadegan *et al.* [6], evaluated and compared the antimicrobial efficacy of 2.5% NaOCl and 2% potassium iodide iodine (IKI) as intracanal disinfectants of the infected canal during single-session endodontic treatments and showed that root canal irrigation with 2.5% NaOCl did not eliminate all the intracanal bacteria; but it had superior efficacy compared to that of 2% IKI.

In general, studies on the antimicrobial efficacy of CHX have demonstrated that CHX has a greater antibacterial effect on microorganisms compared to other antibacterial agents [23]. In a study by Lin *et al.* [24], CHX alone and in combination with CH showed antibacterial efficacy greater than that of CH alone. Moreover, in their clinical trial, Leonardo *et al.* [25] reported that root canal irrigation caused 100% reduction in *Streptococcus mutans* count and 77.78% reduction in anaerobic bacterial count. Bidar *et al.* [26], showed optimal efficacy of 2% CHX for elimination of *E. coli*, *Streptococcus mutans*, *Candida albicans*, *Enterococcus faecalis* and *Lactobacillus* from the root canals.

CH was used in the current study due to its recognized antibacterial and antifungal activity, effects on bacterial biofilm, the synergistic effect between CH and other agents, significant effects on dentin properties and infiltration of hydroxyl ions throughout dentin [27]. 

*E. faecalis* is the dominant isolated microorganism from the infected root canals [28] and due to its high resistance against antimicrobial agents, it has been used in many studies [29]. The *E. faecalis* strains may lodge into dentinal tubules and stay alive for long periods of time as a single microorganism in the root canal system without being supported by other strains [30]. 

In the current study, culture technique was employed to assess the antimicrobial effects of root canal irrigation and disinfection regimens. The culture technique is reliable to detect and count viable bacteria following antimicrobial treatments. In some other studies, a positive association has been reported between negative culture results and optimal therapeutic outcome [3, 31]. However, in this technique, about half the endodontic microbiota are not cultured [32]; moreover, during the process of culture, bacterial virulence factors, which are more important that the quantity of viable bacteria in apical periodontitis, are not detected [19]. Furthermore, negative results of microbial culture do not necessarily indicate sterility of the environment [33] and are rather indicative of the fact that the bacterial count has decreased to the level not detectable by the conventional culture techniques [34]. 

In teeth with periapical lesions and a vital pulp, single session root canal treatment is ideal due to the low count of intracanal bacteria and given that the treatment is performed aseptically, there would be no reason not to accomplish the treatment within a single session. However, for teeth with necrotic pulps and periapical lesions, there are some concerns regarding single session biomechanical preparation and subsequent obturation of the root canal since the adequate quality of root canal cleaning and disinfection cannot be ensured [35]. 

Based on the results of the current study, application of a mixture of CH and CHX as an intracanal medicament for two weeks following root canal irrigation with 6% NaOCl caused a significant reduction in aerobic, anaerobic and *E. faecalis* colony counts in the root canal. Thus, the success rate would probably increase if a two-session treatment protocol is followed for teeth with periapical lesions. Considering these reductions in bacterial counts, teeth with periapical lesions can be treated primarily using the two-session treatment protocol, which is applied for retreatment of teeth with periapical lesions in order to have predictable results. However, further studies are required to evaluate the efficacy of this treatment protocol against other bacterial strains. Patient satisfaction may also increase if a long single-session treatment is replaced with two shorter sessions with more predictable results.

## Conclusion

Primary treatment of necrotic teeth with endodontic lesions can be completed in two sessions with intracanal medicaments like CH and CHX with predictable and favorable results.
